# Focusing on Comorbidity—A Novel Meta-Analytic Approach and Protocol to Disentangle the Specific Neuroanatomy of Co-occurring Mental Disorders

**DOI:** 10.3389/fpsyt.2021.807839

**Published:** 2022-01-18

**Authors:** Lydia Fortea, Anton Albajes-Eizagirre, Yuan-Wei Yao, Edu Soler, Norma Verdolini, Alexander O. Hauson, Adriana Fortea, Santiago Madero, Aleix Solanes, Scott C. Wollman, Maria Serra-Blasco, Toby Wise, Steve Lukito, Maria Picó-Pérez, Christina Carlisi, JinTao Zhang, PingLei Pan, Álvar Farré-Colomés, Danilo Arnone, Matthew J. Kempton, Carles Soriano-Mas, Katya Rubia, Luke Norman, Paolo Fusar-Poli, David Mataix-Cols, Marc Valentí, Esther Via, Narcis Cardoner, Marco Solmi, Jae I. Shin, Eduard Vieta, Joaquim Radua

**Affiliations:** ^1^Institut d'Investigacions Biomèdiques August Pi I Sunyer (IDIBAPS), Barcelona, Spain; ^2^Centro de Investigación Biomédica en Red de Salud Mental (CIBERSAM), Instituto de Salud Carlos III, Madrid, Spain; ^3^Department of Medicine, University of Barcelona, Barcelona, Spain; ^4^Department of Education and Psychology, Freie Universität Berlin, Berlin, Germany; ^5^Einstein Center for Neurosciences Berlin, Charité - Universitätsmedizin Berlin, Berlin, Germany; ^6^Berlin School of Mind and Brain, Humboldt-Universität zu Berlin, Berlin, Germany; ^7^Bipolar and Depressive Disorders Unit, Hospital Clinic, Barcelona, Spain; ^8^Clinical Psychology PhD Program, California School of Professional Psychology, San Diego, CA, United States; ^9^Department of Psychiatry, University of California San Diego, La Jolla, CA, United States; ^10^Fundació Clínic per a la Recerca Biomèdica (FCRB), Barcelona, Spain; ^11^Psychiatric and Psychology Service, Hospital Clinic, Barcelona, Spain; ^12^Schizophrenia Unit, Hospital Clinic, Barcelona, Spain; ^13^Department of Psychiatry and Forensic Medicine, Autonomous University of Barcelona, Barcelona, Spain; ^14^Department of Psychology, Abat Oliba CEU (“Centro de Estudios Universitarios”) University, Barcelona, Spain; ^15^Programa E-Health ICOnnecta't, Institut Català d'Oncologia, Barcelona, Spain; ^16^Department of Neuroimaging, Institute of Psychiatry, Psychology, and Neuroscience, King's College London, London, United Kingdom; ^17^Department of Child and Adolescent Psychiatry, Institute of Psychiatry, Psychology, and Neuroscience, King's College London, London, United Kingdom; ^18^Live and Health Sciences Research Institute (ICVS), University of Minho, Braga, Portugal; ^19^ICVS/3B's, PT Government Associate Laboratory, Braga, Portugal; ^20^Clinical Academic Center - Braga, Braga, Portugal; ^21^Division of Psychology and Language Sciences, University College London, London, United Kingdom; ^22^State Key Laboratory of Cognitive Neuroscience and Learning and IDG/McGovern Institute for Brain Research, Beijing Normal University, Beijing, China; ^23^Department of Neurology, Department of Addictive Behavior and Addiction Medicine, Central Institute of Mental Health, Affiliated Yancheng Hospital of Southeast University, Yancheng, China; ^24^Medical Faculty Mannheim, University of Heidelberg, Mannheim, Germany; ^25^Department of Psychological Medicine, Institute of Psychiatry, Psychology, and Neuroscience, King's College London, London, United Kingdom; ^26^Department of Psychiatry and Behavioral Science, College of Medicine and Health Sciences, United Arab Emirates University (UAEU), Al Ain, United Arab Emirates; ^27^Department of Psychosis Studies, Institute of Psychiatry, Psychology & Neuroscience, King's College London, London, United Kingdom; ^28^Psychiatry and Mental Health Group, Neuroscience Program, Bellvitge Biomedical Research Institute (IDIBELL), L'Hospitalet de Llobregat, Barcelona, Spain; ^29^Department of Psychobiology and Methodology of Health Sciences, Universitat Autònoma de Barcelona, Barcelona, Spain; ^30^Department of Psychiatry, University of Michigan, Ann Arbor, MI, United States; ^31^The Social and Behavioral Research Branch, National Human Genome Research Institute, National Institute of Health, Bethesda, MD, United States; ^32^Early Psychosis: Interventions and Clinical-Detection (EPIC) Lab, Department of Psychosis Studies, Institute of Psychiatry, Psychology, London, United Kingdom; ^33^Department of Brain and Behavioral Sciences, University of Pavia, Pavia, Italy; ^34^Outreach and Support in South London (OASIS) Service, South London and Maudsley NHS Foundation Trust, London, United Kingdom; ^35^Department of Clinical Neuroscience, Center for Psychiatry Research, Karolinska Institutet, Stockholm, Sweden; ^36^Stockholm Health Care Services, Stockholm County Council, Stockholm, Sweden; ^37^Child and Adolescent Psychiatry and Psychology Department, Hospital Sant Joan de Déu, Barcelona, Spain; ^38^Child and Adolescent Mental Health Research Group, Institut de Recerca Sant Joan de Déu, Barcelona, Spain; ^39^Mental Health Department, Hospital Universitari Parc Taulí, Institut d'Investigació i Innovació Parc Taulí (I3PT), Sabadell, Spain; ^40^Department of Psychiatry, University of Ottawa, Ottawa, ON, Canada; ^41^Department of Mental Health, The Ottawa Hospital, Ottawa, ON, Canada; ^42^Clinical Epidemiology Program, Ottawa Hospital Research Institute, Ottawa, ON, Canada; ^43^Department of Pediatrics, Yonsei University College of Medicine, Seoul, South Korea

**Keywords:** meta-analysis, magnetic resonance imaging (MRI), seed-based d mapping (SDM), gray matter (GM), mental disorder, comorbidity, medication

## Abstract

**Background:**

In mental health, comorbidities are the norm rather than the exception. However, current meta-analytic methods for summarizing the neural correlates of mental disorders do not consider comorbidities, reducing them to a source of noise and bias rather than benefitting from their valuable information.

**Objectives:**

We describe and validate a novel neuroimaging meta-analytic approach that focuses on comorbidities. In addition, we present the protocol for a meta-analysis of all major mental disorders and their comorbidities.

**Methods:**

The novel approach consists of a modification of Seed-based d Mapping—with Permutation of Subject Images (SDM-PSI) in which the linear models have no intercept. As in previous SDM meta-analyses, the dependent variable is the brain anatomical difference between patients and controls in a voxel. However, there is no primary disorder, and the independent variables are the percentages of patients with each disorder and each pair of potentially comorbid disorders. We use simulations to validate and provide an example of this novel approach, which correctly disentangled the abnormalities associated with each disorder and comorbidity. We then describe a protocol for conducting the new meta-analysis of all major mental disorders and their comorbidities. Specifically, we will include all voxel-based morphometry (VBM) studies of mental disorders for which a meta-analysis has already been published, including at least 10 studies. We will use the novel approach to analyze all included studies in two separate single linear models, one for children/adolescents and one for adults.

**Discussion:**

The novel approach is a valid method to focus on comorbidities. The meta-analysis will yield a comprehensive atlas of the neuroanatomy of all major mental disorders and their comorbidities, which we hope might help develop potential diagnostic and therapeutic tools.

## Introduction

Authors have reported potential brain anatomical abnormalities for different mental disorders since the 1980s (1). At present, the neuroscientific community has enough data (thousands of studies) to create an atlas of these abnormalities, but this is not yet a reality due to the heterogeneity in the findings across studies investigating the same disorder. For instance, a meta-analysis of structural brain alterations of social anxiety disorder (SAD) found that studies presented contradictory findings, such as increases and decreases in gray matter (GM) volume in the hippocampus and other brain regions (2). Similarly, whereas several meta-analyses had reported significant SAD-related abnormalities in GM in the amygdala-hippocampal, prefrontal, and parietal regions (3–5), an ENIGMA study only found a significant larger GM volume in the right putamen (6). Another example could be the case of obsessive-compulsive disorder (OCD) neuroanatomical findings, where although the abnormalities of the corticostriatal-thalamocortical circuits have been consistently reported (7–9), recent evidence has been accumulating to other regions outside these circuits with less agreement among meta- and mega-analysis reports (7, 10, 11). Moreover, the exact direction of increases and decreases in GM volume in certain areas, such as the orbitofrontal cortex (OFC), has been unclear (12). For example, some reports show reduced GM in bilateral (13) or right (14) OFC, whereas there are also studies reporting increased bilateral (15) or left (16) OFC.

The found heterogeneity may be partly related to the use of magnetic resonance imaging (MRI) devices with varying field strengths (17) and head coils (18) or to the techniques or software used to process the images (18, 19). In addition, several clinical parameters might moderate the findings, such as the age at disease onset and the duration of disease (20, 21), the current age (22), symptom heterogeneity (23), the gender distribution (24), the medication status (25, 26), or clinical stage (27). For example, in a meta-analysis of attention-deficit hyperactivity disorder (ADHD) (28), we found that samples with more medicated patients showed less decreased GM volume in the right caudate.

Another relevant but little explored source of heterogeneity may be the varying presence of comorbidities, which are the rule in psychiatry (29). About half of people with mental disorders have more than one comorbid mental disorder (30, 31). Some authors claim that “there are no patients without comorbidity” (32). We must acknowledge that some studies exclude individuals with specific comorbid mental disorders. Still, this exclusion is frequently limited to very few entities such as psychosis and substance use disorders. Similarly, we must acknowledge that some comorbidities are conceptually impossible, e.g., a patient with bipolar disorder (BD) cannot be diagnosed with comorbid major depressive disorder (MDD), but again, the list of impossible comorbidities is limited. Therefore, most patients included in a study of a given mental disorder might also have other mental disorders. For example, in a meta-analysis of neuroimaging studies in OCD, patients had comorbid depression or anxiety disorders in 65% of the studies. The percentage of patients with comorbid disorders reached 50% (for depression) and 85% (for anxiety disorders) in some included studies (33). This common presence of abnormalities from other mental disorders might thus confound the results of case-control studies.

Relevantly, previous studies have found that some brain abnormalities associated with different mental disorders are non-specific. For example, in several meta-analyses, we observed similar decreases of GM volume in the anterior cingulate/medial frontal cortex in disorders as different as psychosis, anxiety disorders (AD), ADHD, and autism spectrum disorders (ASD) (33–37). Similarly, an ENIGMA study reported a high similarity of brain structural abnormalities between MDD, BD, OCD, and schizophrenia (38, 39). We fully acknowledge that some of these non-specific abnormalities may be transdiagnostic, i.e., associated with two or more mental disorders. However, there is also the possibility that some others are related to the confounding effects of comorbidities.

This study aims to describe and validate a novel neuroimaging meta-analytic approach that focuses on comorbidities and presents the protocol for a meta-analysis of all major mental disorders and their potential comorbidities. We exemplify this protocol for voxel-based morphometry (VBM) studies investigating GM volume differences between patients with mental disorders and healthy controls. However, it could be similarly applied to any neuroimaging modality compatible with SDM (e.g., functional MRI or diffusion tensor imaging). Specifically, we will meta-analyze brain abnormalities from different disorders with different comorbidities, with a single meta-linear model (though separately for children/adolescents and adults). This analysis will yield an MRI-based atlas that dissects the specific brain anatomical abnormalities of each mental disorder and comorbidity. In complementary analyses and depending on data availability reported in the studies, we will explore the potentially confounding or moderating effects of age, sex, medication, age of onset, duration of illness, and symptom severity.

## Methods and Analysis

### The Novel Approach

The new approach is conceptually novel, but it involves only a minor modification of the “seed-based d mapping-permutation of subject images” (SDM-PSI) (www.sdmproject.com) (11, 40–42), an already validated and widely used brain imaging meta-analytic method (43–51). The main advantage of this method is that it directly tests whether there are differences between patients and controls, rather than conducting indirect tests such as whether peaks tend to converge in some regions more than in others (52).

It first creates maps of the lower and upper bounds of possible effect sizes for each study based on the available statistical information and the anisotropic covariance between adjacent voxels (53). Second, it uses maximum likelihood estimation techniques to impute several effect sizes maps for each study, assuming that the effect size follows a truncated normal distribution within the lower and upper bounds. Third, it fits a random-effects meta-analytic linear model separately for each voxel. Fourth, it combines the meta-analytic maps resulting from the different imputations using Rubin's rules. Finally, it conducts a permutation test to yield threshold-free cluster enhancement (TFCE)-based (54) familywise error rates (FWER, i.e., corrected *p*-values).

In this paper, we will first describe the new concept, then report the small methodological changes, and finally report a validation of the approach using simulations.

#### Description

Commonly, the primary (random-effects meta-analytic) linear model of a meta-analysis is just a (weighted) mean:


$$\begin{array}{l} Y_{i}=\beta +\varepsilon_{i} \end{array}$$

where *Y*_*i*_ is the effect size of the *i*th study, β is the meta-analytic effect size, and ε_*i*_ is the residual for the *i*^th^ study. It is important to remember that this model is conducted separately for each voxel.

In some previous meta-analyses, we attempted to control for comorbidity via meta-regression (covariation) by the percentage of patients with a comorbid disorder:


$$\begin{array}{l} Y_{i}=\beta_{A}+\beta_{AB-A}X_{i,AB}+\varepsilon_{i} \end{array}$$

where the intercept β_*A*_ is the effect size for patients with only disorder A, the coefficient β_*AB*−*A*_ is the difference between patients with both disorders and patients with only the disorder A, and *X*_*i,AB*_ is the proportion of patients with both disorders in the *i*^th^ study.

However, these attempts had two relevant limitations. First, β_*AB*−*A*_ (the difference between patients with both disorder A and disorder B and patients with only disorder A) mixed the effects of disorder B and the effects of the comorbidity AB. Thus, we could not know which part of β_*AB*−*A*_ was shared by any patient with disorder B (with or without disorder A) and which part of β_*AB*−*A*_ was specific to those patients with both disorder A and disorder B. For instance, imagine that patients with only disorder A show decreased amygdala, patients with only disorder B show decreased cerebellum, and patients with both disorder A and disorder B show decreased amygdala, cerebellum, and prefrontal cortex. The decrease in the cerebellum is shared by any patient with disorder B, and the reduction in the prefrontal cortex is specific to those with both disorder A and disorder B, but β_*AB*−*A*_ would mix both abnormalities. Second, we could only include studies on disorder A in which a variable proportion of patients had disorder B. Conversely, we could not include studies on disorder B in which a variable proportion of patients had the disorder A, with the subsequent loss in precision.

Here, we propose to use the following model to study two disorders A and B:


$$\begin{array}{l} Y_{i}=\beta_{A}X_{iA}+\beta_{B}X_{iB}+\beta_{AB}X_{iAB}+\varepsilon_{i} \end{array}$$

where β_*A*_ is the effect size for patients with only disorder A, *X*_*iA*_ is the proportion of patients with disorder A in the *i*^th^ study, β_*B*_ is the effect size for patients with only disorder B, *X*_*iB*_ is the proportion of patients with disorder B in the *i*^th^ study, β_*AB*_ is the effect size of the comorbidity AB, and *X*_*iAB*_ is the proportion of patients with both disorders in the *i*^th^ study.

This model overcomes the limitations of the previous attempts because it separates the effects of disorder B and the effects of the comorbidity AB and can accept both studies on disorder A and studies on disorder B because it treats all disorders equally. Indeed, we can extend the model to as many disorders and comorbidities as wished. Still, considering the complexity of the analysis and the likely poor reporting of co-occurring comorbidities, we will only consider pairs of comorbid mental disorders that are possible (e.g., anxiety and MDD) and have been studied by at least ten studies.

#### Validation

To validate the novel approach, we simulated 64 studies on disorder A, 64 on disorder B, and 64 on disorder C, with varying levels of comorbidity, and then meta-analyzed them using the novel approach. The reason to simulate 64 studies for each disorder is that we simulated eight levels of comorbidity (including no comorbidity) for each of the two comorbid disorders, e.g., for disorder A, we simulated eight levels of comorbid disorder B X 8 levels of comorbid disorder C.

**Figure 1:**
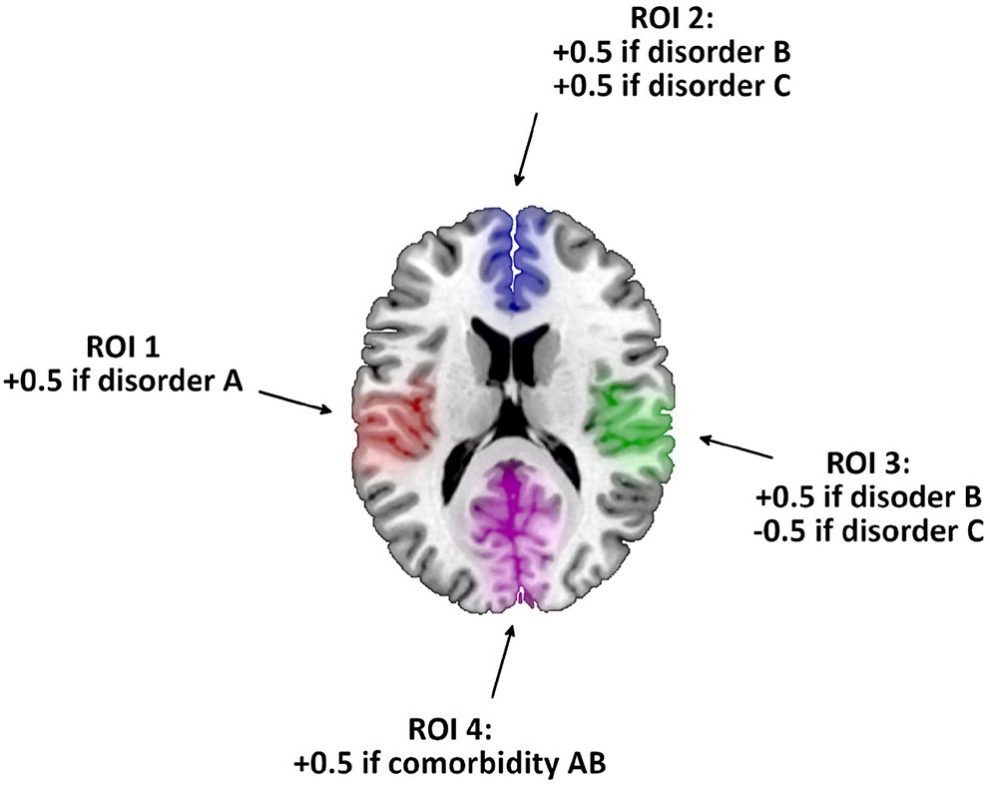
Rules for creating the gray matter maps of a simulated patient. After creating a map using white noise, we added or subtracted 0.5 in the colored brain regions of interest (ROI) depending on the disorders he/she had.

Specifically, we first simulated for each study that a varying proportion of the patients had one of the other disorders or both. We then created the subjects' gray matter maps as white noise following a standard normal distribution. Still, for each patient, we added or subtracted 0.5 in four brain regions depending on the disorders he/she had and the rules in Figure 1. We thus created abnormalities with a medium effect size (Cohen's d around 0.5). Finally, we conducted the voxelwise *t*-test between patients and controls to derive the study t-map.

To meta-analyze the studies' t-maps with the novel SDM-PSI approach (with default values and defining statistical significance as TFCE permutation-based FWER < 0.05), we modeled:


$$\begin{array}{l} Y_{i}=\beta_{A}X_{iA}+\beta_{B}X_{iB}+\beta_{C}X_{iC}+\beta_{AB}X_{iAB}+\beta_{AC}X_{iAC}+\beta_{BC}X_{iBC} \end{array}$$

For example, imagine that disorder A was OCD, disorder B was MDD, and disorder C referred to anxiety disorders. Studies on OCD would be coded as *X*_*iA*_ = 1, *X*_*iAB*_ = [proportion of patients with comorbid MDD], and *X*_*iAC*_ = [proportion of patients with comorbid anxiety disorders]. Studies on MDD would be coded as *X*_*iB*_ = 1, *X*_*iAB*_ = [proportion of patients with comorbid OCD], and *X*_*iBC*_ = [proportion of patients with comorbid anxiety disorders]. Finally, studies on anxiety disorders would be coded as *X*_*iC*_ = 1, *X*_*iAC*_ = [proportion of patients with comorbid OCD], and *X*_*iBC*_ = [proportion of patients with comorbid MDD]. Although studies seldom report the proportion of patients with multiple comorbidities (e.g., in studies on OCD, the proportion of patients with both comorbid MDD and anxiety disorders), our new approach could easily account for them if reported. Meta-analysts should add a regressor for each reported combination of comorbid mental disorders.

For comparison purposes, we also conducted three meta-regressions using the previous SDM-PSI approach (again, with default values and defining statistical significance as TFCE permutation-based FWER < 0.05):


$$\begin{array}{l} Y_{i}=\beta_{A}+\beta_{AB-A}X_{iAB}+\beta_{AC-A}X_{iAC}\\ Y_{i}=\beta_{B}+\beta_{AB-B}X_{iAB}+\beta_{BC-B}X_{iBC}\\ Y_{i}=\beta_{C}+\beta_{AC-C}X_{iAC}+\beta_{BC-C}X_{iBC} \end{array}$$

#### Results of the Validation

**Figure 2:**
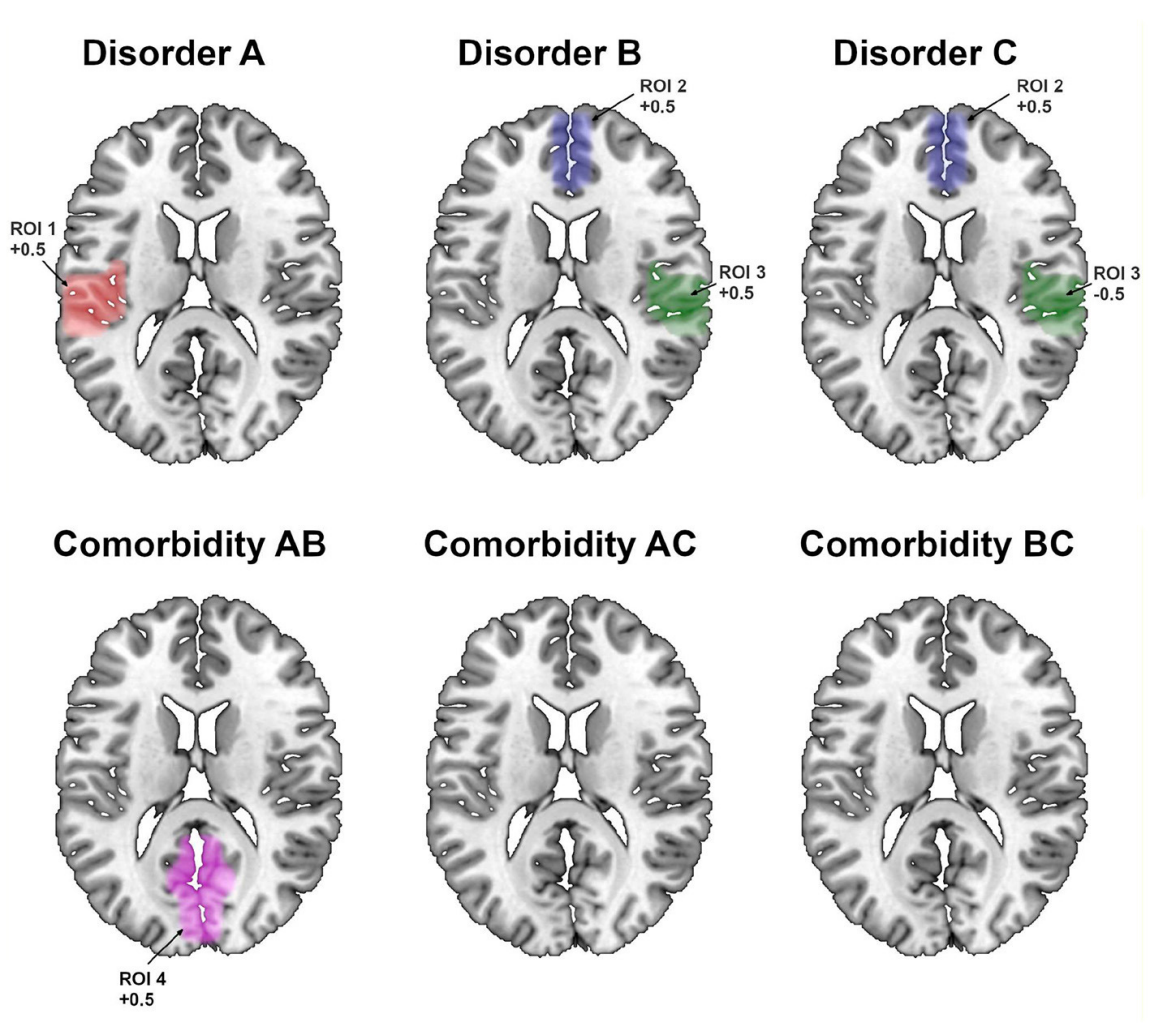
Regions showing statistically significant effects using the novel SDM-PSI approach. ROI, region of interest.

The novel SDM-PSI approach detected all the simulated abnormalities with the correct effect size and did not yield any falsely positive findings (Table 1, left column; and Figure 2).

**Table 1:** Results of the novel approach validation.

	**Simulated factual data**	**Novel SDM-PSI approach**	**Previous SDM-PSI approach**		
			**Main disorder is A**	**Main disorder is B**	**Main disorder is C**
Disorder A	ROI 1, *g* +0.5	*β_*A*_* = ROI 1, *g* +0.5	*β_*A*_* = ROI 1, *g* +0.5 ROI 2, *g* +0.4 ROI 4, *g* +0.2	β__*AB*_−B_ = ROI 1, +0.5 ROI 4, +0.5	β__*AC*_−C_ = ROI 1, *g* +0.5
Comorbidity AB	ROI 4, *g* +0.5	*β_*AB*_* = ROI 4, *g* +0.5	β__*AB*_−A_ = ROI 4, *g* +0.5 ROI 2, *g* +0.4 ROI 3, *g* +0.5		(Not studied)
Disorder B	ROI 2, *g* +0.5 ROI 3, *g* +0.5	*β_*B*_* = ROI 2, *g* +0.5 ROI 3, *g* +0.5		*β_*B*_* = ROI 2, *g* +0.7 ROI 3, *g* +0.3	β__*BC*_−C_ = ROI 2, *g* +0.4 ROI 3, *g* +0.5
Comorbidity BC	(None)	*β_*BC*_* = (None)	(Not studied)	β__*BC*_−B_ = ROI 2, g +0.5 ROI 3, g −0.5	
Disorder C	ROI 2, *g* +0.5 ROI 3, *g* −0.5	*β_*C*_* = ROI 2, *g* +0.5 ROI 3, *g* −0.5	β__*AC*_−A_ = ROI 2, g +0.5 ROI 3, g −0.5		*β_*C*_* = ROI 2, *g* +0.7 ROI 3, *g* −0.3
Comorbidity AC	(None)	*β_*AC*_* = (None)		(Not studied)	(See Disorder A)

“*g”, average Hedges' g of the voxels within the cluster of statistical significance; ROI, region of interest*.

**Figure 3:**
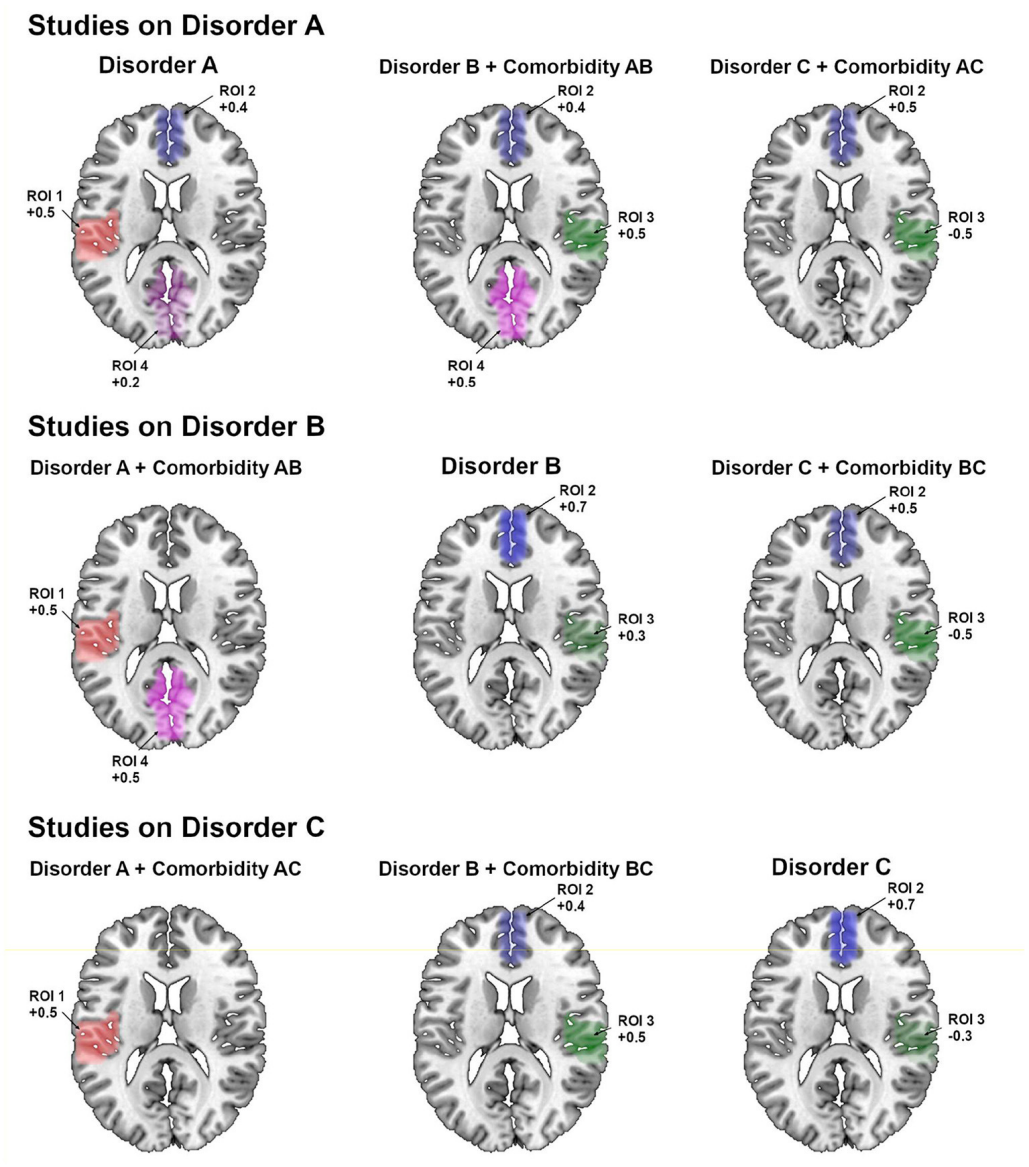
Regions showing statistically significant effects using the previous SDM-PSI approach (separate results for meta-analysis of studies on disorder A, meta-analysis of studies on disorder B, and meta-analysis of studies on disorder C). ROI, region of interest.

The previous SDM-PSI reported a list of findings very similar to the simulated factual data (Table 1, right columns; and Figure 3). However, the results showed the two main limitations we had expected. First, they mixed the abnormalities of the comorbid disorder and the comorbidity. For instance, in the meta-regression using studies on disorder A, the coefficient β_*AB*−*A*_ mixes the anomalies simulated for disorder B and comorbidity AB. We acknowledge that this limitation could be potentially overcome by looking at the meta-regression using studies on disorder B. However, this strategy would be confusing in this example because we would still not know whether abnormality in the region of interest (ROI) 4 is due to disorder A or comorbidity AB. The second limitation was a slight loss of accuracy, as shown by that some Hedges' *g* are slightly different from 0.5, and there are a few falsely positiveresults.

Therefore, while the novel SDM-PSI approach does not invalidate the previous version, it better disentangles the specific abnormalities of comorbid disorders.

Results were similar when we created the simulated data with double error, thus expecting Cohen's d around 0.25 (Table 2). That said, one can expect poorer estimations with smaller effect sizes or in meta-analyses with few studies. Thus, we would not recommend the new approach when the number of studies for each regressor is too small for the expected effect sizes.

**Table 2:** Results of the novel approach validation after creating the simulated data with double error.

	**Simulated factual data**	**Novel SDM-PSI approach**	**Previous SDM-PSI approach**		
			**Main disorder is A**	**Main disorder is B**	**Main disorder is C**
Disorder A	ROI 1, *g* +0.25	*β_*A*_* = ROI 1, *g* +0.25	*β_*A*_* = ROI 1, *g* +0.26 ROI 2, *g* +0.23 ROI 4, *g* +0.11	β__*AB*_−B_ = ROI 1, +0.23 ROI 4, +0.27	β__*AC*_−C_ = ROI 1, *g* +0.25
Comorbidity AB	ROI 4, *g* +0.25	*β_*AB*_* = ROI 4, *g* +0.25	β__*AB*_−A_ = ROI 4, *g* +0.23 ROI 2, *g* +0.22 ROI 3, *g* +0.24		(not studied)
Disorder B	ROI 2, *g* +0.25 ROI 3, *g* +0.25	*β_*B*_* = ROI 2, *g* +0.23 ROI 3, *g* +0.26		*β_*B*_* = ROI 2, *g* +0.35 ROI 3, *g* +0.15	β__*BC*_−C_ = ROI 2, *g* +0.29 ROI 3, *g* +0.25
Comorbidity BC	(None)	*β_*BC*_* = (None)	(Not studied)	β__*BC*_−B_ = ROI 2, g +0.25 ROI 3, g −0.26	
Disorder C	ROI 2, *g* +0.25 ROI 3, *g* −0.25	*β_*C*_* = ROI 2, *g* +0.24 ROI 3, *g* −0.24	β__*AC*_−A_ = ROI 2, g +0.20 ROI 3, g −0.31		*β_*C*_* = ROI 2, *g* +0.37 ROI 3, *g* −0.15
Comorbidity AC	(None)	*β_*AC*_* = (None)		(not studied)	(see Disorder A)

“*g”, average Hedges' g of the voxels within the cluster of statistical significance; ROI, region of interest*.

## Protocol for the -Meta-Analysis

We pre-registered this protocol to PROSPERO (CRD42021245098).

### Design

Meta-regression of case-control VBM studies of GM volume abnormalities in all major mental disorders. The dependent variable will be the brain anatomical differences between patients and controls in a voxel. The independent variables will be the percentages of patients with each mental disorder and each pair of potentially comorbid mental disorders.

### Systematic Search

With few exceptions (see below), we will include all whole-brain VBM studies in any mental disorder listed in the mental, behavioral, or neurodevelopmental disorders classification of ICD-11 (International Classification of Diseases 11th Revision). Note that we will use ICD-11 to select the major disorders to investigate. Still, as we clarify later, we will include studies using any standard clinical assessment beyond ICD (e.g., DSM). Our search will have two steps.

### First Step: Search and Inclusion of Meta-Analyses

In the first step, we will search for the most recent SDM meta-analysis (if any) in the PubMed and Scopus databases for each mental disorder listed in ICD-11 classification (excluding nicotine use disorder, substance-induced specific disorders, neurocognitive disorders, and mental or behavioral disorders associated with pregnancy, childbirth, or the puerperium). The keywords will be the mental disorder (e.g., “major depression,” “anxiety disorders,” “bipolar disorders,” etc.) AND (“meta-analysis”) AND (“voxel-based morphometry” OR “VBM” OR “gray matter” OR “grey matter”). We will first screen all the results by the title/abstract and afterward by full-text reading.

The inclusion criterion will be meta-analyses of studies that employed VBM to investigate whole-brain GM volume differences between patients with the above disorders and healthy controls. The exclusion criterion will be meta-analyses from which we can include <10 studies even after adding new studies as described in the second step. We will select the most recent meta-analysis conducted with SDM if more than one meta-analysis meets our inclusion/exclusion criteria. Suppose the inclusion/exclusion criteria of a meta-analysis led to the exclusion of studies that we would include according to our second-step study inclusion/exclusion criteria (see next). In that case, we will look for these potentially includable studies (e.g., a meta-analysis may have excluded studies in children/adolescents while we will include them). Conversely, suppose the inclusion/exclusion criteria of a meta-analysis led to the inclusion of studies that we would exclude according to our second-step inclusion/exclusion criteria. In that case, we would exclude these studies (e.g., a meta-analysis may have included studies with fewer than 10 participants per group while we will exclude them). Suppose during our search, a new meta-analysis is published after we have included a meta-analysis for the same mental disorder. In that case, we will include both analyses (but we will include the duplicated studies only once.

## Second Step: Search and Inclusion of Individual Studies

In the second step, we will search in PubMed and Scopus databases for the studies published since the search date of the selected meta-analysis. The keywords used in this search will be [Title/Abstract]: (selected mental disorder) and (“VBM” OR “morphometry” OR “voxel-based” OR “voxelwise” OR “gray matter” OR “grey matter”). We will first screen all the results by the title/abstract and then by full-text reading.

Inclusion criteria will be: (1) studies reporting whole-brain regional GM volume differences between individuals with the included mental disorders, diagnosed by standard clinical assessments (DSM or ICD), and matched healthy controls; (2) employing VBM to conduct the comparisons, (3) reporting the peaks of the clusters of statistically significant voxels or null findings, or availability of statistical parametric map; (4) using a constant statistical threshold throughout the whole gray matter; (5) published as peer-reviewed original articles in English in indexed journals. Exclusion criteria will be: (1) sample size smaller than 10 participants in either the patient or the control group; (2) no case-control comparisons; (3) disorders' subtypes with a known organic origin (e.g., pediatric autoimmune neuropsychiatric disorders associated with streptococci); (4) coordinates of the peaks of the clusters cannot be obtained after contacting the authors (unless maps are available, in which case we will not need the coordinates); (5) ROI or small volume correction (SVC) analyses; (6) ANOVA analysis without whole-brain t-test *post hoc* analyses; (7) case reports, conferences abstracts, editorials, non-scientific letters, and research protocols; (8) duplicated datasets (we will only include the largest sample size); (9) studies that only analyze the correlation of GM volume with any other measure or that use GM volume features to predict diagnosis unless they specify an additional VBM case-control comparison in the abstract. Special cases will be: (1) longitudinal studies: we will only include the baseline comparison; (2) studies reporting different subgroup analyses: we will include the combined analysis of all subgroups if available. Otherwise, we will include them as different studies if they use different control groups and provide demographic and clinical data for both subgroups separately. If they share the control group, we will divide the control sample size between the number of subgroups.

Two researchers will conduct the systematic search independently, and we will resolve any discrepancies by consensus with a third researcher.

### Data Collection

For each study separately, we will extract the sample sizes, demographic and clinical data, methodologic details, and the original statistical parametric map (when available) or the coordinates and *t*-values (or equivalent statistics when available) of the peaks of the clusters of statistically significant voxels (or null findings).

Demographic data will include age distribution (mean and standard deviation) and percentage of males and females.

Clinical data will consist of the percentages of patients with different mental disorders, the percentage of patients receiving each medication group (antipsychotics, antidepressants, anxiolytics -other than hypnotic-, mood stabilizers, and stimulants), the severity of the primary disorder assessed by standard measures [e.g., Hamilton Depression Rating Scale (HDRS) (55)], the age of onset or illness duration of the primary disorder (mean and standard deviation), and the different subtypes for the primary disorder as reported in the included meta-analyses (e.g., inattentive, hyperactive, or combined type for ADHD, type I or II for BD, etc.).

Methodological details will include the pre-processing analysis software (e.g., FSL, SPM) and their version, stereotactic space (e.g., MNI, Talairach space, or MNI coordinates converted to Talairach using the old Brett transform), and the statistical significance threshold (e.g., FWER < 0.05). For studies reporting peaks obtained using two or more whole-brain statistical significance levels (e.g., uncorrected *p* < 0.001 and corrected FWER *p* < 0.05), we will include all peaks obtained using the less conservative threshold. We will also record the information required for the quality assessment (see below).

Given the magnitude of data, we will store them in a database and a well-organized file system with automatic daily backups.

### Quality Assessment

We will use the Newcastle-Ottawa Scale (NOS) for case-control studies to assess each study's quality (56). The NOS assesses three characteristics of the studies: the selection of the study groups, the comparability of the groups, and the ascertainment of the exposure for case-control studies. The “selection of the study groups” evaluates the adequate definition of case and control, as well as the representativity of the cases (e.g., selection of all eligible cases with the outcome of interest over a defined period, in a defined catchment area or hospital, etc.), and the controls (community controls or hospital controls). The “comparability of the groups” evaluates if researchers matched cases and controls and/or adjusted for confounders (e.g., age, sex, handedness). Here, statements of no differences between groups or non-statistically significant differences are insufficient for establishing comparability. Finally, we will not evaluate the “ascertainment of the exposure for case-control studies” because both groups underwent a structural MRI in our studies.

We will also assess how much demographic or clinical data each independent study reports.

### Imputation of Missing Comorbidity Data

Not all studies report the percentage of patients with specific comorbid disorders. For instance, in the meta-analysis of OCD mentioned earlier, 12% of studies had not excluded comorbid MDD but did not report how many patients had this diagnosis. To impute these unreported data, we will assume they are missing at random. In other words, the proportion of patients with no information about comorbid MDD should follow a similar distribution than in studies reporting this information.

The proportion of patients with a comorbid disorder likely follows a zero-inflated distribution. For example, the percentage of patients with MDD might follow some statistical distribution, but this distribution probably has excess zeroes due to the studies that excluded patients with MDD. However, as fitting zero-inflated distributions with the small data available would be unfeasible, we will use a more straightforward, distribution-free approach. Specifically, the imputation will consist of assigning to each study not reporting the proportion of patients with comorbid MDD, the proportion from another random OCD study. Thus, for example, we may estimate that the missing proportion in a given study is the same as in the study by van den Heuvel et al. (57), or the same as in the study by Pujol et al. (58), or the same as in any other random study (including studies that excluded MDD).

We will repeat these imputations 50 times. We want to remark that his number is commonly considered more than adequate for multiple imputation (Rubin recommended 3 to 10 imputations (59). Additionally, we have checked that the histogram of the imputed proportions of patients with MDD is similar to the histogram of known proportions of patients with MDD after only ten imputations and nearly identical after twenty imputations.

These imputations will be conducted separately for each comorbid disorder. Thus, for instance, in studies with OCD, we will impute comorbid MDD and comorbid anxiety disorders separately.

### Statistical Analyses

We will carry out the data pre-processing and the statistical analysis with the SDM-PSI 6.21 software (https://www.sdmproject.com/) (11, 40–42). We will conduct two independent analyses, one for adults and one for children/adolescents.

The pre-processing of statistical parametric maps is the straightforward conversion into images of effect sizes. For studies with only peak information available, the pre-processing consists of estimating the 3D images of the lower and higher bounds of potential effect sizes. The software will later impute the effect sizes multiple times within these bounds.

We will include all major mental disorders in one single linear model described earlier. Then, we will estimate the effects related to each disorder and comorbidity by testing different contrasts within the model. The steps will be those of standard SDM-PSI (11, 40–42) unless otherwise specified:

1. Estimation of the 3D images of maximum likely effect sizes for each model coefficient.
2. Multiple imputation of the study 3D images of effect size adding spatially realistic noise to the expected effect size according to the estimated distribution within the bounds. Following SDM-PSI default parameters, we will conduct this process 50 times, resulting in 50 imputed datasets covering the imputations' uncertainty.
3. Separately for each imputation dataset, random-effects meta-linear model. The dependent variable will be the effect size of the voxel. The independent variables will be the percentages of patients with each mental disorder and the percentages of patients with each pair of potentially comorbid disorders (as far as they involve at least ten studies). In case that at least studies reported the percentage of patients with more than three or more comorbid disorders (e.g., OCD, MDD, and anxiety), we will also include these percentages as an independent variable in the model.
4. Using Rubin's rules, combination of the meta-analytic 3D images of effect size from the different imputation datasets.

To assess the statistical significance, the software converts the 3D image of z-values into a 3D image of threshold-free cluster enhancement (TFCE) statistics and finds the *p*-value of the TFCE statistics using a Freedman-Lane-based permutation test (60). We will consider statistically significant those voxels with family-wise error-rate (FWER, i.e., corrected *p*-values) <0.05. For comprehensive reporting, we will also publish supplementary results using significance thresholds of FWER < 0.01, uncorrected *p* < 0.001, and uncorrected *p* < 0.005.

In the complementary analyses, we will add additional independent variables to the linear model to explore potential interactions with mean sample age and percentage of males and control the medication's possible confounding effects. Depending on our final dataset, we will try to assess the effects of the age of onset or illness duration and the severity of the primary disorder, as reported in the original studies. Furthermore, we will perform a subgroup analysis excluding those disorders for which we cannot safely collect whether they are comorbidities for other disorders (e.g., we expect that we will not have information on comorbid personality disorders in many studies).

We will use the *I*^2^ statistic to quantify heterogeneity and conduct meta-regression by the standard error [similar to an Egger-test (41)] to detect potential publication bias. Conventionally, *I*^2^ values above 50% are interpreted as an indication of significant heterogeneity (61).

## Discussion

This paper first presents and validates a neuroimaging meta-analytic approach that focuses on comorbidities in mental disorders. Then, using simulations, we show that the new method may detect all GM volume differences with the correct effect size and without falsely positive findings. Finally, we describe the protocol for a meta-analysis of all major mental disorders and their comorbidities, separately for adult and pediatric groups. We will also assess the potentially confounding effects of medication, age of onset or illness duration and the symptom severity of the primary disorder, and the moderator effects of sex on GM volume.

We broadly expect some findings according to previous literature, though a significant part of these findings might change due to the improvements of the new approach. For example, for chronic schizophrenia, previous meta-analyses have detected reduced GM volume in the bilateral insula/ superior temporal gyrus, dorsal, and rostral anterior cingulate cortex (ACC) / medial frontal gyrus, and the thalamus (62, 63). Similarly, for the first episode of psychosis, we expect a reduced GM volume in the right dorsal ACC and the right posterior insula/superior temporal gyrus (35, 62, 63). In OCD, previous meta-analyses have detected both increased GM volumes, mainly located in subcortical regions (e.g., bilateral putamen, left cerebellum, and left hippocampus), and decreased GM volumes, located primarily on prefrontal and cingulate areas (e.g., bilateral ACC/ventromedial prefrontal cortex, bilateral inferior frontal gyrus) (9, 33, 34). For BD and MDD, previous meta-analyses have detected a commonly reduced GM volume in the medial prefrontal system and ACC, regions strongly implicated in mood regulation (64). However, smaller hippocampus and parahippocampal gyrus volumes have been more reported in MDD (26, 37, 65–69). For ADHD, previous meta-analyses have reported a reduced GM volume in ventromedial orbitofrontal cortex/ventromedial prefrontal cortex/rostral ACC, and the right basal ganglia/anterior and posterior insula (34, 70). For ASD, previous meta-analyses have reported reduced GM volume in dorsal ACC/dorsomedial prefrontal cortex, left cerebellum, and increased GM volume in the left middle superior anterior lobe and middle frontal gyrus (33, 70). For anxiety disorders, previous meta-analyses have reported a reduced GM volume in the right ventral ACC and inferior frontal gyrus (71). These areas have been primarily reported in panic disorder, together with the prefrontal cortex (72, 73). However, some of these findings may have been influenced by comorbidity. Thus, we need to conduct the new meta-analysis to know which results remain, which do not, and which had not been detected due to the confounding effects of comorbidities.

We hope that this improved atlas of the anatomical localization of the brain abnormalities associated with each mental disorder will help improve our understanding of their physio-pathological processes. In addition, we hope that this atlas could be the base for developing MRI-based diagnostic tools that help earlier diagnoses and, therefore, more targeted treatments. For instance, when complex psychotic symptoms hamper the assessment of other symptoms needed for the diagnosis, the “opinion” of an MRI-based diagnostic tool could provide timely extra information to establish an affective vs. non-affective diagnosis and thus a more focused treatment earlier. Or similarly, in depressed individuals at risk of manic shift, the “opinion” of an MRI-based diagnostic tool may help the clinician better evaluate the probability of bipolar vs. unipolar disorder and thus design a more personalized preventive strategy. Indeed, we have found elsewhere that diagnostic labels are among the variables that best predict the future recurrence of an episodic disorder.

Better knowledge about the disorder-specific abnormalities could also increase the efficacy of therapies to modify specific brain regions' activity [e.g., deep brain stimulation or non-invasive brain stimulations such as repetitive transcranial magnetic stimulation (74)]. Improved knowledge about the spatial distribution of these abnormalities may help localize the brain targets better. Indeed, previous studies have already shown how the efficacy of such therapies depends on the exact position of the brain target (75).

We acknowledge that the novel approach has several limitations. The first relates to the debatable nosology of current mental disorders, based on clinical consensus rather than known biological underpinnings. We know, for example, that major psychiatric disorders share some genetic risk factors, and there are high percentages of comorbidity and diagnostic change. However, this does not mean that there are no disorder-specific brain correlates. As noted above, diagnostic labels are among the best predictors of future outcomes, highlighting their clinical relevance. The second relates to the commonly poor reporting of some comorbid disorders in the literature and the subthreshold disorder-specific symptoms that we will not consider due to the complexity of the analysis and the expected large amount of missing data. For instance, studies may check for schizophrenia but not for personality disorders. For this reason, we will conduct subgroup analyses excluding the disorders under-reported as comorbidities. A third limitation is that, as stated earlier, some studies may not report the proportion of patients with specific comorbidities. Thus, we will have to use multiple imputation. We will use a simplistic imputation algorithm without considering whether the proportion of patients with a given comorbid disorder may depend on the age or symptom severity of the sample. We preferred this simple algorithm because we anticipate that we would not be able to collect the necessary data for robustly using more complex imputation algorithms. Another limitation is that, as in any other meta-analysis, a potential drawback may be the heterogeneity across studies. Considering comorbidity, age, sex, and medication, we aim to explain the heterogeneity more than previous meta-analyses, but we anticipate that there will still be unexplained heterogeneity. A significant source of heterogeneity may be due to differences in the MRI equipment (e.g., varying field strength or head coils) and acquisition parameters (76) and the VBM processing method employed by the different studies, such as software and version, normalization, statistical correction, or the size of the smoothing kernel (19). Another relevant source of heterogeneity may be different subject-specific artifacts such as head motion (77), body mass index (78), drops in signal-to-noise ratio due to susceptibility artifacts, the symptom severity of the disorders, or the different phases present in some disorders (e.g., the various episodes in BD) (79, 80). Also, we will only study those mental disorders for which a meta-analysis has already been published and examined by at least ten studies. Last but not least, we must highlight that even when, for simplicity, we talk about GM volume abnormalities, we should more appropriately refer to differences in T1-MRI signal, given that the acquired MRI data are not a direct measure of brain structure (81).

## Author Contributions

LF, AA-E, and JR conceived the study, modified the SDM-PSI software, and validated the novel approach. All authors participated in the redaction of the manuscript, including minor or major modifications of the protocol.

## Funding

This work was supported by several grants from the Instituto de Salud Carlos III - Subdirección General de Evaluación y Fomento de la Investigación: Research Project Grant (PI19/00394 to JR), Miguel Servet Research Contract (CPII19/0009 to JR), and the PFIS contract (FI20/00047 to LF). NV thanks the BITRECS project, which has received funding from the European Union's Horizon 2020 research and innovation programme under the Marie Skłodowska-Curie Grant Agreement No 754550 and from La Caixa Foundation (ID 100010434), under the agreement LCF/PR/GN18/50310006.

## Conflict of Interest

NV has received financial support for CME activities and travel funds from the following entities (unrelated to the present work): Angelini, Janssen-Cilag, Lundbeck, Otsuka. EVia is a co-investigator in a Janssen-Cilag, S.A., clinical trial with the esketamine molecule (unrelated to the present work). KR received a grant from TAKEDA pharmaceutical for another project and consulting fees from Lundbeck and Supernus (unrelated to the present work). The remaining authors declare that the research was conducted in the absence of any commercial or financial relationships that could be construed as a potential conflict of interest.

## Publisher's Note

All claims expressed in this article are solely those of the authors and do not necessarily represent those of their affiliated organizations, or those of the publisher, the editors and the reviewers. Any product that may be evaluated in this article, or claim that may be made by its manufacturer, is not guaranteed or endorsed by the publisher.
